# Explosive regeneration and anamorphic development of legs in the house centipede *Scutigera coleoptrata*

**DOI:** 10.1186/s12983-024-00544-0

**Published:** 2024-09-19

**Authors:** Iulia Barutia, Andy Sombke

**Affiliations:** 1 Department of Evolutionary Biology, Integrative Zoology, University of Vienna, Djerassiplatz 1, 1030 Vienna, Austria; 2 Institute for Zoology and Evolutionary Research, Animal Physiology, Friedrich-Schiller-University Jena, Erbertstrasse 1, 07743 Jena, Germany; 3 Max-Planck-Institute for Chemical Ecology, Hans-Knöll-Straße 8, 07745 Jena, Germany; 4 Center for Anatomy and Cell Biology, Cell and Developmental Biology, Medical University of Vienna, Schwarzspanierstrasse 17, 1090 Vienna, Austria

**Keywords:** Arthropoda, Chilopoda, Morphology, Histology, MicroCT, Blastema, Appendotomy

## Abstract

**Background:**

Regenerating legs is advantageous for arthropods as their appendages exhibit crucial functional specializations. Many arthropods possess a ‘preferred breakage point’, where the appendage is most likely to break and where regeneration likely to occur, however, different taxa exhibit different levels of regenerative potential. Centipede appendage regeneration is categorized as 'progressive' or 'explosive'. In the later, the appendage is fully regenerated after one molt. This term was used for house centipedes that frequently lose their long legs. We chose *Scutigera coleoptrata* as a model to comprehensively investigate the process of leg appendotomy and regeneration as well as compare it with leg development in anamorphic instars.

**Results:**

The trochanter exhibits a preferred breakage point. Internally, it houses a three-layered diaphragm that effectively seals the lumen. In case of leg loss, the wound is quickly sealed. The epidermis detaches from the cuticle and muscles of the coxa get compacted, giving sufficient space for the regenerating leg. A blastema forms and the leg then grows in a coiled manner. The regenerating leg is innervated and syncytial muscles form. If the leg is lost in an early intermolt phase, progression of regeneration is slower than when a specimen is closer to the next molt. Instars of house centipedes can simultaneously develop and regenerate legs. The legs develop laterally on the posterior segments under the cuticle. As opposed to regeneration, the progression of leg development always follows the same temporal pattern throughout the entire intermolt phase.

**Conclusion:**

Several factors are of major significance in house centipede leg regeneration. First, the ease with which they lose legs: the diaphragm represents an efficient tool for appendotomy. Moreover, the functional extension of the coxa provides space for a leg to be regenerated in. Lastly, the genetic predisposition allows them to regenerate legs within one molting cycle. This “package” is unique among land arthropods, and to this degree rare in marine taxa. Furthermore, observing leg regeneration and anamorphic leg development in parallel suggest that regeneration is most likely an epiphenomenon of development, and the differences are a requirement for the novel context in which re-development occurs.

## Introduction

Regeneration is a widespread phenomenon observed in various animal species. However, the process of regeneration can vary significantly among different animals and even within different tissues in the same organism [1–3]. In Arthropoda, a taxon defined by its sclerotized exoskeleton and articulated legs (arthropodia) [4], it can occur in juveniles and adults. Certain species can e.g. regenerate posterior and postanal body parts, such as the telson in Xiphosura and some decapod Crustacea [5, 6], or even entire parts of the trunk in the exceptional cases of Pycnogonida [7–9]. However, most arthropod regeneration is limited to appendages such as antennae, mouthparts and legs [1]. Uniramous arthropod legs typically consist of five to seven podomeres [10], and parts of it are often lost during an arthropod's lifetime due to predation or inter- and intraspecific conflicts. The ability to replace lost legs is advantageous for arthropods as their appendages exhibit crucial functional specializations beyond locomotion, including prey capture, communication and copulation, respiration, and reception of various stimuli [10–12].

Regeneration of arthropod appendages is influenced and constrained by multiple factors, including the type and stage of postembryonic development, the appendage type lost, the intermolt phase at the time of injury, and the location of the breakage [1, 13]. Hormones, especially ecdysteroids, also play a role in the regeneration process, and their levels—changing throughout the molting cycle—determine the onset of regeneration [14]. If an appendage is lost after a certain ‘critical point’ during the molting cycle, regeneration will be delayed until after the molt [15–17]. Moreover, many arthropod taxa possess a ‘preferred breakage point’ (PBP) or ‘autotomy plane’ where the appendage is most likely to break after injury and where regeneration is most likely to occur. The loss of an appendage at the PBP will be further on referred to as appendotomy (see nomenclature in [1]). It is important to point out that appendage loss can also occur without a PBP, and that regeneration is not dependent on its presence. Scorpions, for instance, can regenerate their pretarsus regardless of the level at which the appendage was removed. In contrast, harvestmen (Opiliones) lose their legs at a PBP to escape predators, yet they lack the ability to regenerate them altogether [1, 18, 19].

Different arthropods exhibit different levels of regenerative potential [13]. Regeneration can be (1) absent, as in most hemipterans and in harvestmen; (2) poor, where the regenerated leg is structurally abnormal or only a small portion of it is regenerated, as seen in scorpions [20]; (3) good, where a structurally normal leg can be regenerated under specific conditions, such as in black widow spiders [21]; or (4) very good, when a structurally normal leg is regenerated under most conditions, observed in juvenile blattodeans, or decapod and amphipod crustaceans [22]. On the other hand, Verhoeff [23] categorized appendage regeneration based on the speed and degree to which arthropods can regenerate: (1) "progressive” regeneration, where the appendage requires multiple molting cycles to become structurally normal, approximate the original size, and in some cases regain functionality [24], and (2) "explosive" regeneration, where the appendage has its original size and functionality after a single molt.

**Fig. 1:**
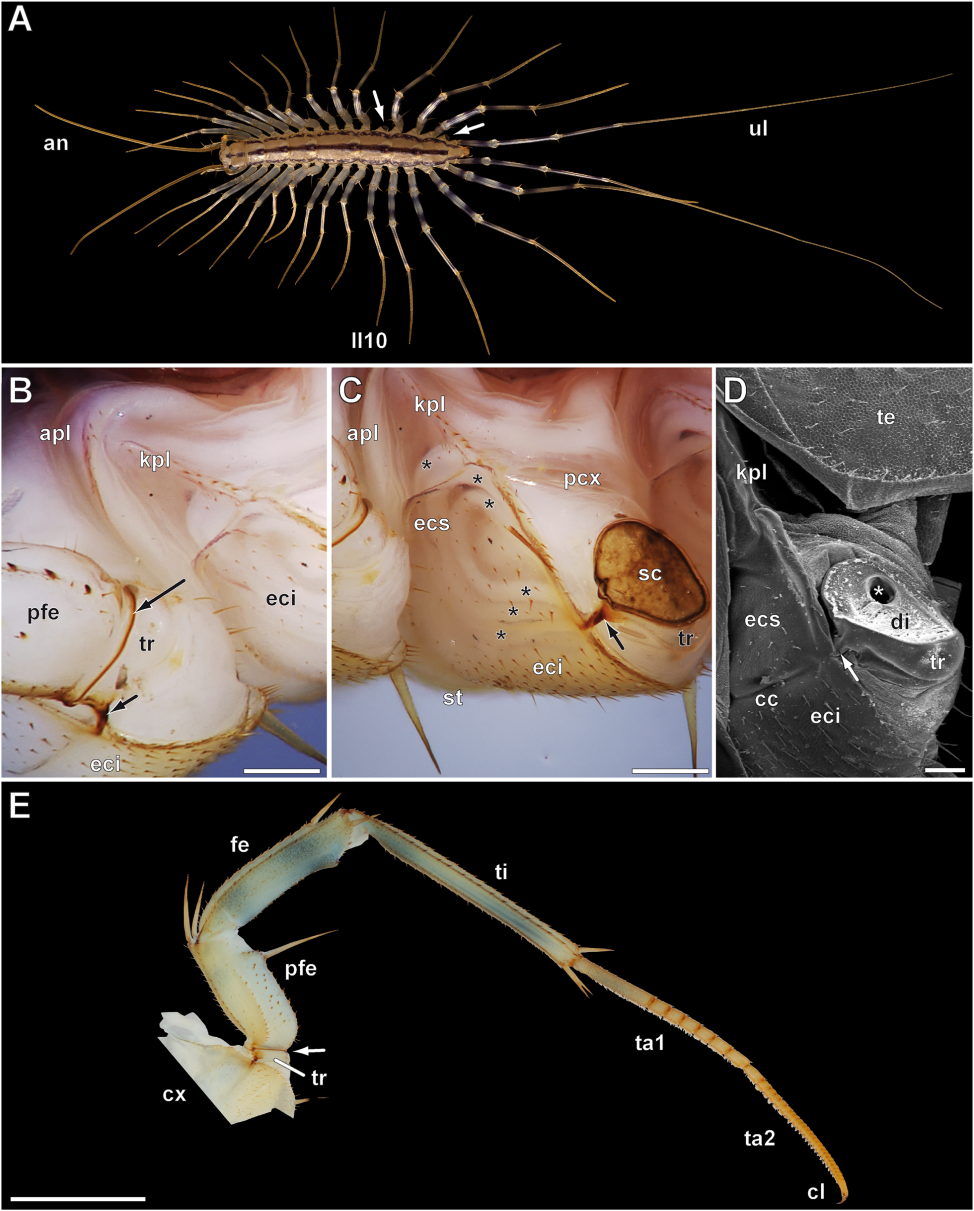
External morphology of *Scutigera coleoptrata*.** A** Adult female, dorsal view, ca. 2.5 cm body length. Antennae (partially broken and thus shorter) and ultimate legs point anteriad and posteriad, respectively. The length of locomotory legs increases from anterior to posterior. From the 14 pairs of locomotory legs (ultimate leg pair = 15), the right legs 10 and 14 are missing (arrows). **B** Lateral view on pleural elements of trunk segments 10 and 11 as well as the proximal locomotory leg 10. The boundary between prefemur and trochanter (= preferred breakage point) exhibits a stronger sclerotization (long arrow). The anterior joint (or anterior pivot) between coxa and trochanter is strongly sclerotized (short arrow) and continues internally as costa coxalis (see also Fig. 1D). Scale bar = 250 µm. **C** Lateral view on pleural and leg elements of trunk segment 10 seven days after appendotomy. A melanized scab covers the wound. Note the sclerotized ring (= preferred breakage point) as well as the sclerotized anterior trochanter-coxa joint (arrow). The regenerating leg is visible coiled inside the coxa (asterisks), reaching up to the katopleura. Scale bar = 300 µm. **D** SEM image of coxa and trochanter of a locomotory leg with pleural elements. The leg was detached at the PBP after fixation to reveal the intact distal fibrous layer of the diaphragm. The diaphragm seals the lumen of the trochanter only leaving passages (asterisk) for the leg nerve, the hemolymph channel and the flexor tibiae trochanteris (compare Fig. 2A, E). Leftovers of hemolymph cover the smaller channel (left below asterisk). The costa coxalis marks the border between eucoxa superior and inferior. Scale bar = 100 µm. **E** Locomotory leg 10 of an adult specimen, view from anterior (note the sclerotized coxa-trochanter joint). The arrow points to the sclerotized ring of the preferred breakage point. Scale bar = 500 µm. Abbreviations: an antenna, apl anopleura, cc costa coxalis, cl pretarsal claw, cx coxa, di diaphragm, eci eucoxa inferior, ecs eucoxa superior, fe femur, kpl katopleura, ll10 locomotory leg 10, pfe prefemur, pcx pleurocoxa, pfe prefemur, sc scab, st sternite, ta1 tarsus 1, ta2 tarsus 2, te tergite, ti tibia, tr trochanter, ul ultimate leg

The term "explosive regeneration” was specifically used by Verhoeff to describe the regeneration ability in house centipedes (Chilopoda, Scutigeromorpha) (Fig. 1A). They are the sister taxon to all other centipedes [25] and possess characteristic features such as compound eyes, unpaired spiracles located dorsally, as well as long and flexible legs [12, 26, 27]. In adults, the 14 pairs of locomotory legs become progressively longer from anterior to posterior. The ultimate pair of legs (the 15th pair) is very long and oriented posteriad (Fig. 1A), presumably serving a pronounced sensory function [12, 28]. Among approximately 100 described species [29], *Scutigera coleoptrata* is the most prevalent and known species in Europe and Northern America. Its distribution peaks in the Mediterranean region, but the species is also found in patches across central Europe, particularly in low-altitude rocky areas with pine forests or urban environments such as basements. The long legs of house centipedes not only contribute to their impressive swiftness of at least half a meter per second [30] (and pers. observ.), but also enable them to entangle and immobilize prey using their lasso-like annulated tarsi. Contrary to other centipede taxa, house centipedes are not adapted for a burrowing lifestyle, although they can easily escape through narrow crevices [31].

Few reports (mostly from the early twentieth century) document instances of regeneration in juvenile and adult centipedes, based on museum specimens or experimental studies (summarized in [1, 32]). Progressive leg and antennal regeneration has been observed in giant and stone centipedes (Scolopendromorpha and Lithobiomorpha), where the regenerated structures tend to be smaller in size [33–38]. In contrast, Scutigeromorpha frequently lose one or more legs in nature, as their long legs can detach very easily at the PBP. The wound is quickly sealed by hemolymph, which dries and hardens on its surface very fast [39]. Cameron [39] and Verhoeff [40] observed that lost legs are fully regenerated within a single molting cycle (explosive regeneration), if appendotomy occurs before the so-called critical point of the molting cycle, approximately seven days before molting in adult specimens. The regenerating legs grow in a spiral manner in the coxa, as Bordage [41] first hypothesized after observing this growth pattern of regenerating legs in Mantodea, Phasmida, Decapoda and Araneae. In *S. coleoptrata*, Demange [42] witnessed regeneration during the examination of a molting specimen, revealing both missing ultimate legs unfolding from under the old cuticle of the coxae.

There is still an overall debate whether adult regeneration is a part of the developmental processes of an animal or a distinct phenomenon independent of development [43–45]. Both processes share many similarities, including regulatory factors and specific gene expression patterns for initial development that also participate in regeneration. Nevertheless, it seems that there are also regulatory networks, which are strictly regeneration-specific [45–47]. *Scutigera coleoptrata* is an ideal model to compare leg development and leg regeneration since this species undergoes anamorphic development: individuals hatch with an incomplete number of segments, and leg-bearing segments develop posteriorly and are added through subsequent molting cycles until the final number of 15 is reached [40, 48, 49]. Bordage [41] initially described the coiled development of legs on the instars' developing segments under the cuticle, while Murakami [48] conducted a more detailed investigation in *Thereuopoda clunifera* (Scutigeromorpha), revealing that the developing legs coil dorsally under the cuticle of the last leg-bearing segment and possess small external leg buds lateroventrally. These newly developed legs are fully functional after molting. In addition, house centipedes have a multi-year lifespan and continue to molt (and regenerate legs) throughout their lives, even when no further segment addition or growth occurs [31].

This study aims to comprehensively investigate the process of leg appendotomy, regeneration and leg development in *S. coleoptrata* using histology, immunohistochemical experiments, and μCT analyses. The mechanism, which allows these animals to appendotomize legs so easily might be associated with their ability to regenerate their legs so efficiently. Understanding the morphology behind these two processes, as well as comparing them in the same individual might give insights into the relation between development and regeneration in arthropod appendages.

## Results

### The preferred breakage point, the diaphragm and the appendotomy of locomotory legs

The long and slender telopodite of the locomotory leg is anchored in the monocondylic coxa, which is surrounded by a flexible, lightly sclerotized membranous cuticle, along with several sclerites (Fig. 1B–E). The trochanter is connected to the coxa by two joints (facilitating the lifting and lowering of the telopodite), one strongly sclerotized anterior joint (Fig. 1B, C; short arrow) and a smaller, hinge-like posterior joint. There are no joints between trochanter and prefemur. All subsequent podomeres have two dorsally positioned joints, including the two flexible and annulated tarsi.

**Fig. 2:**
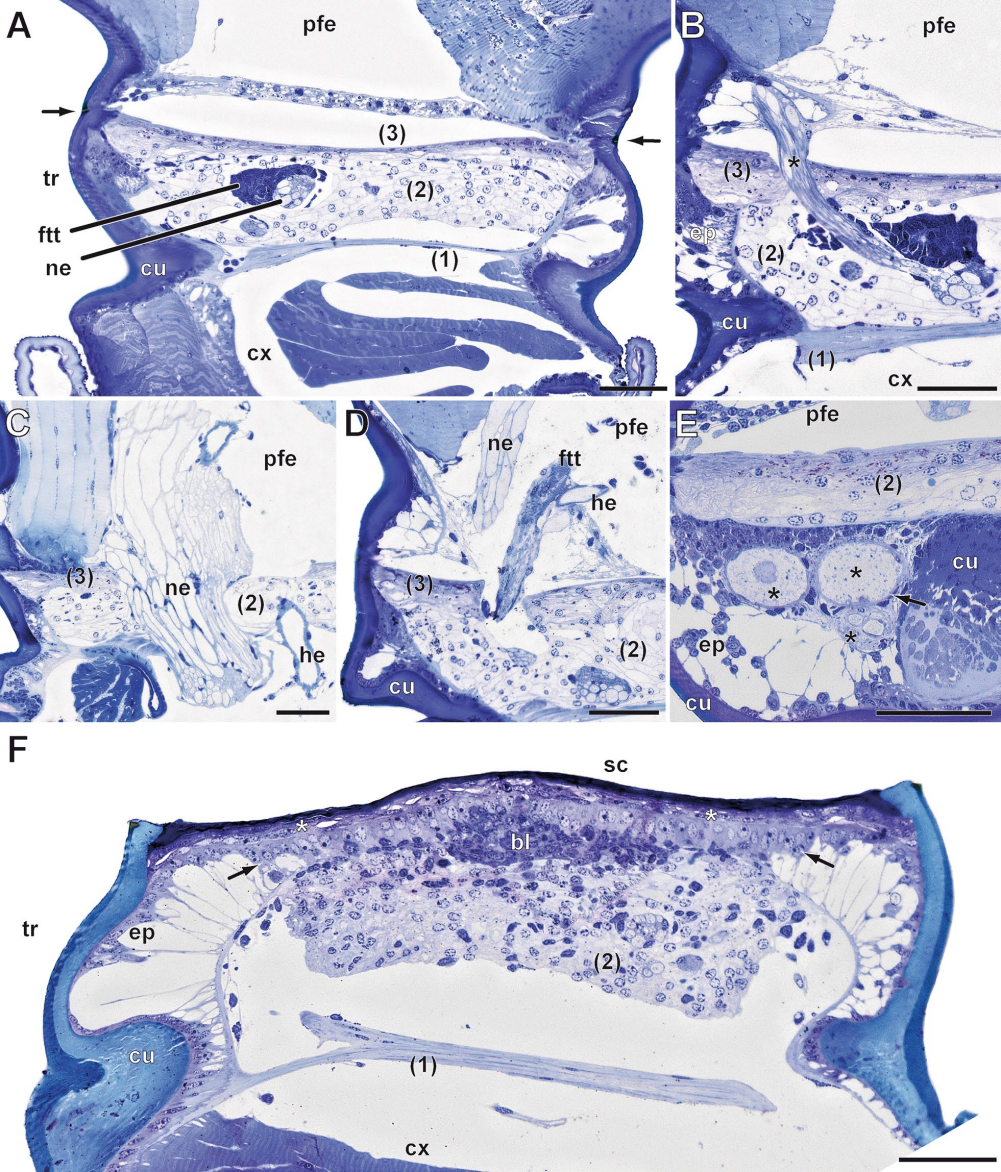
Anatomy of the trochanter and its diaphragm in *Scutigera coleoptrata*. **A** Horizontal section through the proximal locomotory leg. Coxa (bottom) and prefemur (top) house intrinsic musculature. The trochanter is septated by the three transverse layers of the diaphragm. The proximal layer (1) and the distal layer (3) are thin and dense and consist only of few cells and fibers; the medial layer (2) is composed of voluminous cells of connective tissue with characteristic round nuclei featuring little heterochromatin. The leg nerve and the flexor tibiae trochanteris (ftt) penetrate the diaphragm. The ftt only passes the distal layer (3) of the diaphragm, attaching at the apodeme of the anterior coxa-trochanter joint and the proximoventral margin of the tibia [51]. The stronger sclerotized ring of the preferred breakage point is clearly visible (arrows). In the proximal prefemur, another septum of presumably connective tissue is present. Scale bar = 100 µm. **B** Different section level than A, showing the leg nerve (asterisk) penetrating the diaphragm and entering the prefemur. Scale bar = 50 µm. **C** Different aspect of the leg nerve and the hemolymph vessel penetrating the diaphragm. Scale bar = 50 µm. **D** Different aspect of the leg nerve, the hemolymph vessel, and the flexor tibia trochanteris penetrating the diaphragm. Scale bar = 50 µm. **E** Horizontal section through the ventral region of the trochanter. Central (right) cuticle represents the small invagination of the ventral trochanter. Next to it, voluminous neuronal profiles (asterisks; ‘cell complex’ sensu Herbst [52]) surrounded by polymorphic nuclei (arrow; presumably glial cells) are present. Epidermal cells partially possess long processes and build up a sponge-like network. In this region, only the medial layer (2) of the diaphragm is visible. Scale bar = 50 µm. **F** Horizontal section through the trochanter 48 h after appendotomy. The wound is covered by a melanized scab and the dense layer (3) of the diaphragm (asterisks). Epidermal cells migrate under layer (3) (arrows) and close the wound additionally. In between the epidermis and the connective tissue of the diaphragm, characteristic cells with little cytoplasm are present that form the blastema. Scale bar = 50 µm. Abbreviations: bl blastema, cx coxa, cu cuticle, ep epidermis, he hemolymph vessel, ne nerve, ftt flexor tibiae trochanteris, pfe prefemur, sc scab, tr trochanter

Externally, the PBP is easily identifiable by a darker band: the distal, strongly sclerotized margin of the trochanter (distal trochanteral ring) (Fig. 1B; long arrow). The trochanter exhibits specialized anatomical features, which facilitate appendotomy. Internally, it consists of a complex diaphragm composed of three transverse layers: a proximal and a distal fibrous layer encompassing a mass of connective tissue (Fig. 2A–E). The diaphragm effectively seals the lumen of the trochanter, allowing only the leg nerve, a hemolymph vessel, and one small intrinsic muscle to pass through (Fig. 2A–D). The nerve and the hemolymph vessel most likely pass through the same orifice in the distal fibrous layer (Fig. 1D), which probably contracts and closes after appendotomy. The hemolymph vessel originates from the ventral vessel of the trunk [see also 50] and extends laterally towards the leg alongside the nerve, which emerges from the ganglion of the ventral nerve cord. The muscle, flexor tibiae trochanteris, extending from the anteroproximal margin of the trochanter, passes through the distal fibrous layer, and attaches to the proximal tibia [see also 51] (Fig. 2A, B).

Both fibrous layers of the diaphragm are characterized by cells with sparsely distributed elongated nuclei (Fig. 2A–D). They are attached to the trochanter's cuticle by elongated, spindle-shaped epidermal cells (Fig. 2B, D). Within the connective tissue, multiple cells with spherical nuclei are present (Fig. 2A–E). In this region of the diaphragm, the epidermis of the trochanter exhibits a pseudostratified columnar organization, in contrast to the simple cuboidal epithelium that lines the cuticle of other podomeres (Fig. 2B, C). On the ventral side of the trochanter, there are several large neuronal profiles (‘cell complex’ sensu Herbst [52]), surrounded by numerous smaller cells with elongated polymorphic nuclei, possibly glial cells (Fig. 2E). Upon passing through the proximal fibrous layer of the diaphragm, the leg nerve bifurcates, and a smaller branch extends towards this region (not shown). The proximal prefemur exhibits a single transverse layer (Fig. 2A), which likewise seals the telopodite after appendotomy.

In case of leg loss, there is only a drop of hemolymph that covers the wound. After the breakage occurs, the diaphragm slightly bulges outwards, most likely due to an increase in pressure resulting from the closure of the hemolymph channel. The flexor tibiae trochanteris detaches from the trochanter and is lost together with the leg. With no additional structures passing through, the multilayered diaphragm in the trochanter can effectively seal the wound and prevent further hemolymph loss. If an injury occurs distally of the PBP (e.g., femur; Fig. 1E), hemolymph loss is greater, and the animal will nibble the wound and eventually tear off the damaged leg with its mouthparts within a few minutes after injury.

### Wound healing and regeneration of the locomotory leg

**Fig. 3:**
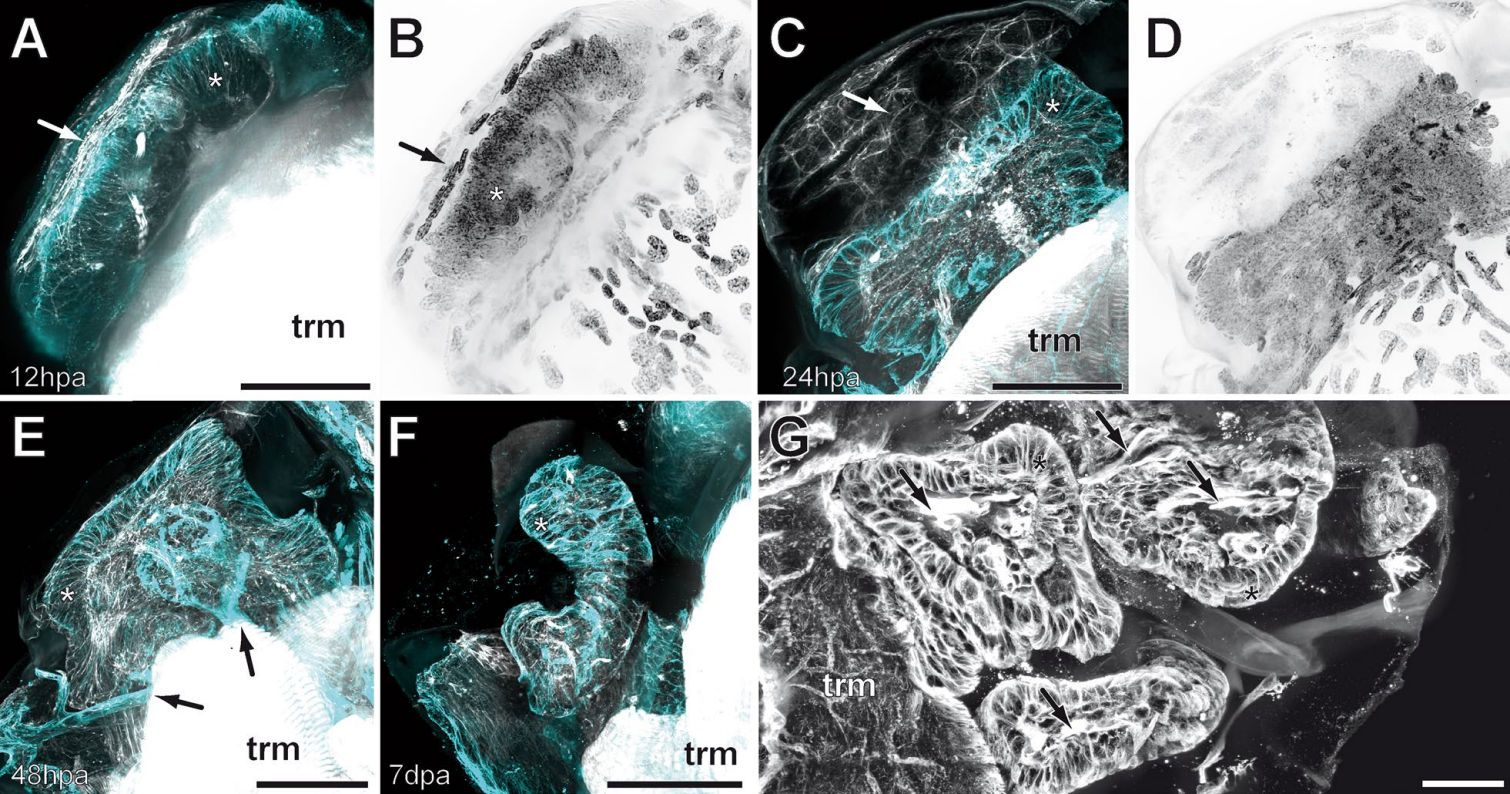
Immunohistochemical experiments in regenerating legs in instar VI of *Scutigera coleoptrata*.** A** Tubulin immunoreactivity (cyan) and phalloidin labeling (white) 12 h after appendotomy. Cells form the trochanteral epidermis migrate under the distal fibrous layer to close the wound internally. Tubulin-ir reveals the close association of cells of the epidermis in a columnar fashion (asterisk), as well as the distal layer of the diaphragm with the densely packed, phalloidin labelled fibers (arrow). Few weakly phalloidin labeled fibers are also present centrally (compare intense labeling of the trunk musculature). **B** Nuclear labeling (black) 12 h after appendotomy (same section as in A) reveals a horizontal zone of cells with more elongate nuclei (arrow) belonging to the distal layer, and the epidermal cells with round nuclei closing the wound (asterisk). **C** Tubulin-ir (cyan) and phalloidin labeling (white) 24 h after appendotomy. Tubulin-ir labeling reveals the highly ordered fashion of the epidermis of the regenerating leg that is continuous with the peripheral epidermis. Centrally, the cells of the blastema are polymorphic and their cytoskeleton is labelled much weaker. Distally, tubulin-ir is absent (arrow). Phalloidin labeling likewise reveals the domelike organization of the epidermis with its columnar organization (asterisk). At least two stronger labelled bundles innervate the central mass. Distally, a network of phalloidin labeled fibers is present (arrow). **D** Nuclear labeling (black) 24 h after appendotomy (same section as in C) reveals that the blastema is a tight aggregation of many cells with little cytoplasm. **E** Tubulin-ir (cyan) and phalloidin labeling (white) 48 h after appendotomy. Tubulin-ir reveals the shaping of the regenerating leg. The columnar epithelium is multilayered (asterisk). Centrally, only weak tubulin-ir is detectable, however, nervous innervation from the ventral nerve cord is present (black arrows). In this section, a second nerve is visible that innervates a region of the posterior coxa. Phalloidin labeling reveals a central structure of interweaving fibers. **F** Tubulin-ir (cyan) and phalloidin labeling (white) seven days after appendotomy. The regenerating leg takes up a distinct tube-like shape with a central lumen. **G** Tubulin-ir (white) ca. 2 weeks after appendotomy. The regenerating leg fills out the coxal lumen and exhibits several loops. The unilayered epithelium (asterisks) encloses a central lumen that contains several muscle fiber bundles (arrows). All scale bars = 50 µm. Abbreviations: trm trunk musculature

**Fig. 4:**
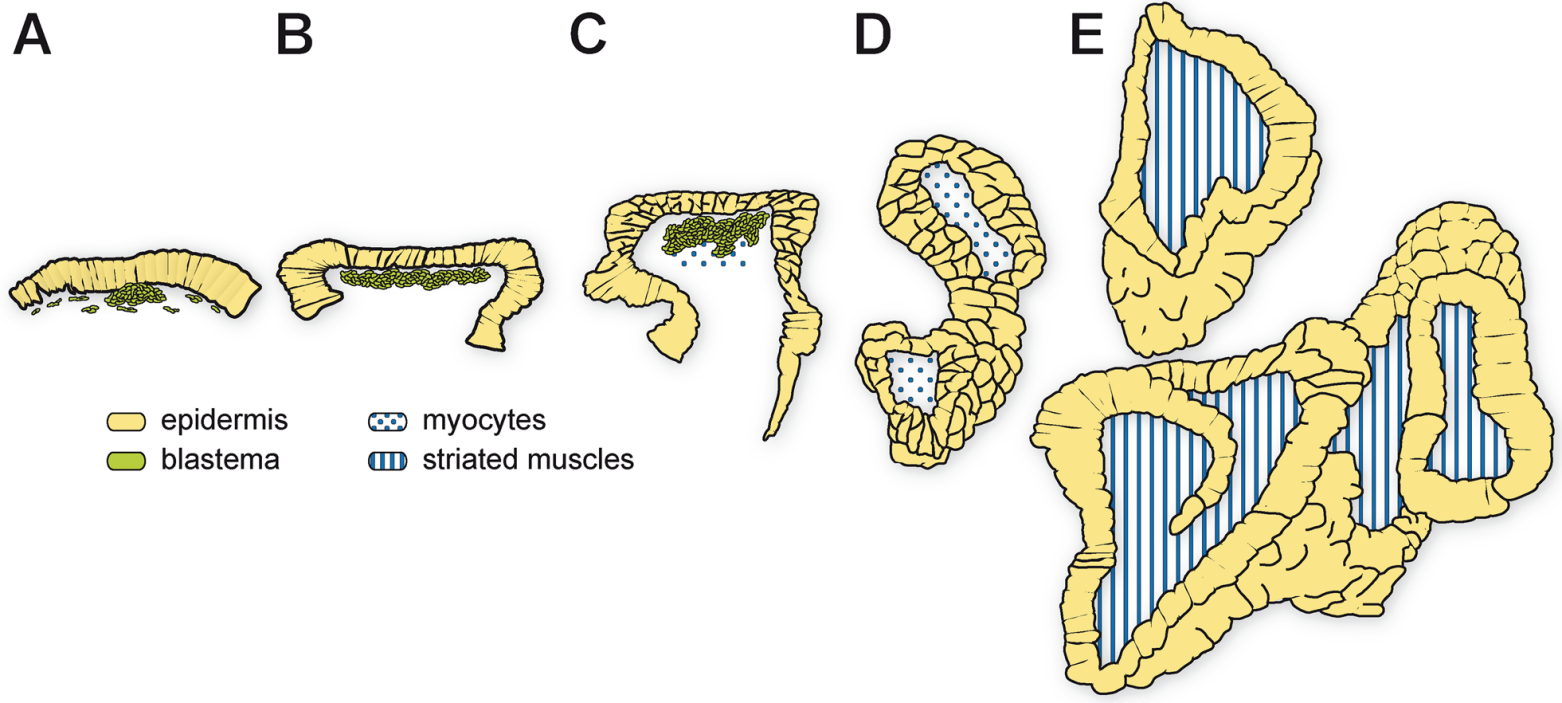
Schematic representation of early adult leg regeneration in *Scutigera coleoptrata*.** A** Early phase, ca. 12 h post appendotomy. The epidermis of the trochanter detaches and migrates over the wound to close it. Underneath the epidermis, the blastema starts to form. **B** The blastema grows and lines the epidermis at the wound site. **C** After ca. 48 h, the epidermis becomes multilayered and bulges out. Within the blastema, myocytes are detectable. **D** While regeneration progresses, the leg elongates, becomes coiled, and has a multilayered epidermis. Internally, myocytes are present. **E** Later phase of regeneration. The leg is coiled within the coxa, the epidermis is unilayered and striated muscles are present

**Fig. 5:**
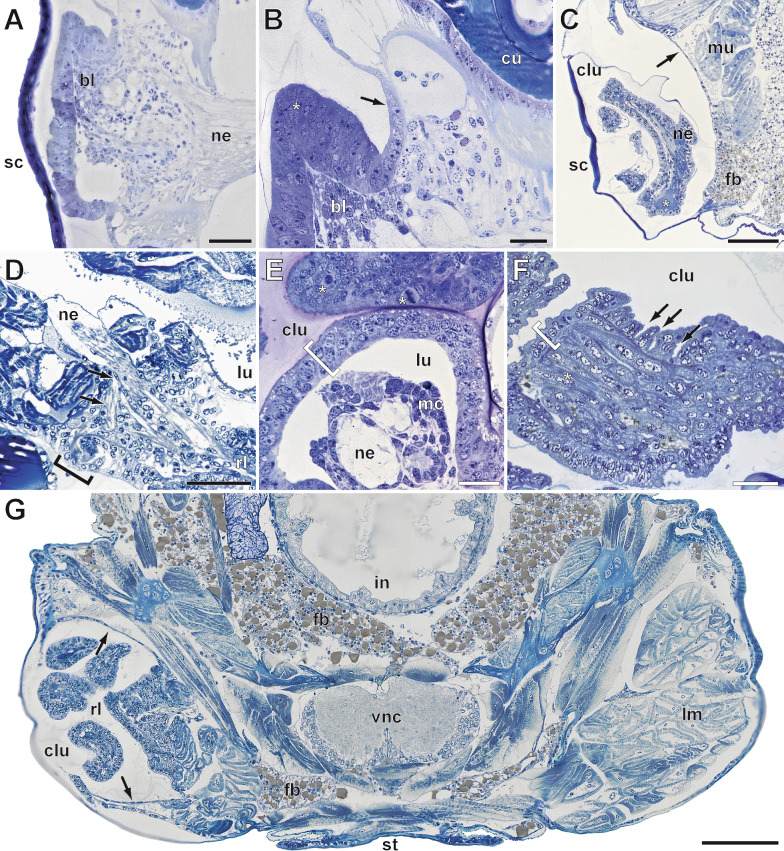
Histological aspects of leg regeneration in *Scutigera coleoptrata*.** A** Early blastema formation 24 h post appendotomy. Under the scab, the epithelium migrates to close the wound and is multilayered. The leg nerve is present. Scale bar = 50 µm.** B** Higher magnification of A. Epidermis of the trochanter (arrow) that detaches from the cuticle. While migrating to close the wound, the epidermis becomes multilayered (asterisk). Underneath, the blastema starts forming. Scale bar = 20 µm. **C** Trochanteral lumen between the cuticle and the epidermis of the body wall (arrow) with regenerating leg at 7 dpa, whose lumen is filled with hemolymph (asterisk), myocytes and the leg nerve. Scale bar = 100 µm. **D** Detail of the nervous innervation of the regenerating leg. The regenerated leg nerve bifurcates at the level of the trochanter (arrows) and also innervates the newly forming trochanteral “cell complex” (bracket). Scale bar = 50 µm. **E** Cross section through the regenerating leg at 7 dpa in early intermolt phase. The epithelium is still multilayered (bracket)**,** with central neural innervation and myocytes. Frequently, mitotic activity is detectable (asterisks). Scale bar = 20 µm.** F** Cross section through the regenerating leg at 7 dpa in late intermolt phase. The epidermis is unilayered (bracket) and forms macro-folds (arrows), which are characteristic for the preparation for molting. The lumen is completely filled with central syncytial muscles bundles. Scale bar = 20 µm. **G** Cross section through the trunk of instar 5 dpa. Compare the musculature of the right locomotory leg coxa and the coxal lumen of the regenerating left locomotory leg. The lumen is lined by the epidermis of the body wall (arrows). Scale bar = 100 µm. Abbreviations: bl blastema, clu coxal lumen, cu cuticle, fb fat body, in intestine, lm locomotory leg musculature, lu leg lumen, mc myocytes, mu musculature, ne nerve, rl regenerating leg, sc scab, st sternite, vnc ventral nerve cord

**Fig. 6.**
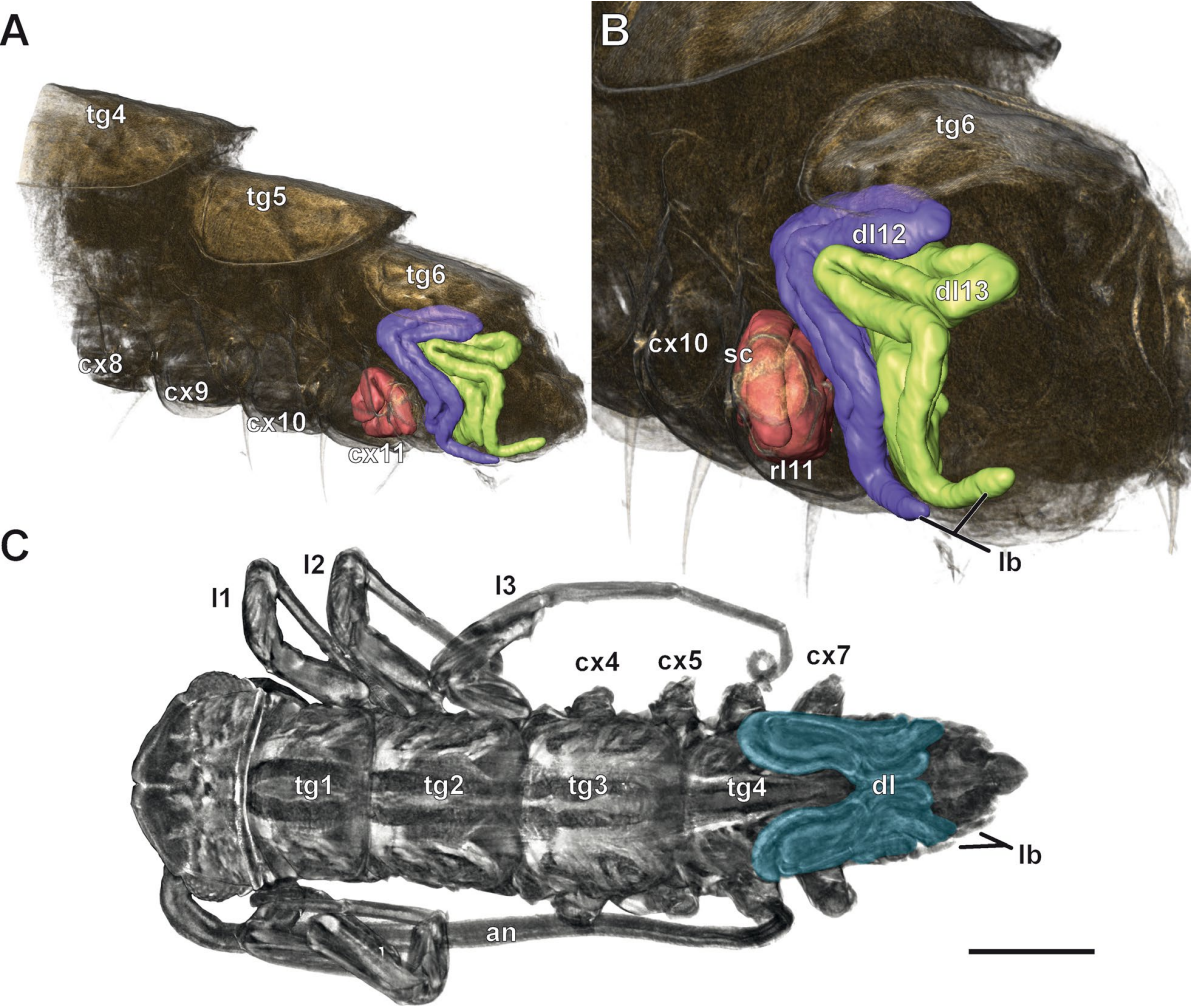
3D visualizations of instar stages III and V of *Scutigera coleoptrata* with regenerating and developing legs (microCT).** A** Instar stage V. Dorsolateral view on the posterior trunk with reconstructed regenerating leg (left leg segment 11) and developing legs (segments 12 and 13). Note that locomotory legs are detached and only coxae are visible and that Scutigeromorpha possess fewer tergites than sternites. **B** Magnified view of the same visualization as in A. The scab of the distal trochanter is clearly visible and the regenerating leg is coiled within the coxa of trunk segment 11. Developing legs are coiled laterally and dorsally under the tergite over the developing segments 12 and 13. The external limb buds of the developing legs are visible ventrally (see also Fig. 7A, D). Note the size difference between the spaces in which the regenerating and developing legs grow. **C** Instar stage III. Dorsal view on the whole animal; some legs are detached. Blue represents the developing legs eight and nine on top of the developing segments. Scale bar = 500 µm. Abbreviations: an antenna, cx coxa, dl developing leg, l leg, lb limb buds, rl regenerating leg, sc scab, tg tergite

After appendotomy, the hemolymph covering the wound quickly dries up and forms a scab. Within 12 h post appendotomy (hpa), the scab undergoes a process of hardening and melanization (Figs. 1C and 2F). The epidermis of the trochanter starts to detach from the cuticle and migrates across the lumen, thus completely closing the wound under the distal fibrous layer (Figs. 2F arrows, 3A, B and 4A). The lumen of the trochanter now is filled with tightly packed cells with round nuclei (Figs. 2F, 3A, B and 4B), which form the blastema. After wound healing, leg regeneration commences only if the appendotomy occurred before the critical point of the molting cycle. In our experiments, the critical point varies among individuals, from approximately two days pre-molt in young instars to seven days in adults. If the appendage loss occurs before this point, the blastema starts to grow and then to differentiate (Figs. 3E, 4B, C and 5A, B), forming the new leg. As the regenerating leg continues to grow in size, the epidermis of the coxa detaches from the cuticle and migrates inwards, toward the body cavity (Fig. 5B, C, G). Simultaneously, the muscles in the coxa become strongly compacted inwards, toward the midline of the body (Fig. 5C, G). This process creates sufficient space inside the coxa for the regenerating leg to grow in a coiled manner (Figs. 5C, G and 6A, B).

First, a multilayered columnar epithelium, continuous with the epidermis of the coxa forms (Figs. 3C and 5A, B). In its lumen, polymorphic cells with very little cytoplasm are tightly packed. In the area between the regenerating leg and the scab, there is a loose network of fibers, and scattered cell nuclei, most likely a connective tissue (Fig. 3C, D). As the leg regenerates, the leg nerve projects into its lumen and myocytes are present (Fig. 5A, C–E). The diaphragm newly forms together with the neuronal profiles next to the ventral epidermis, already innervated by a nerve branching from the leg nerve (Fig. 5D). Mitosis of epidermal cells of the regenerating leg epithelium are often observable (Fig. 5E). As regeneration progresses, the multilayered epidermis transitions into a unilayered structure, and syncytial muscles become striated and form bundles that are innervated by neurites (Figs. 4D, E and 5E *versus* F). In the late stages of regeneration, the unilayered epidermis starts forming macro-folds (Fig. 5F arrows), which are characteristic of an epidermis that secretes a new cuticle in preparation for molting (as described for hexapods [53]).

### Timing of regeneration

The timing and speed of leg morphogenesis, the steps following blastema formation described above, vary depending on the time point of the intermolt phase the animal is in. Legs were removed from different specimens at the same number of days post-molt (dpm) to analyze the onset of regeneration in early intermolt stages (legs removed at 10 dpm) and late intermolt stage (25 dpm), with an average duration of the molting cycle of about 30–40 days. Moreover, legs were removed from the same individuals in further six consecutive days to compare the progression of regeneration between the two time points during the intermolt phase.

In the early intermolt phase, the progression of regeneration is considerably slower and the regeneration is not completed in the timeframe of observation (7 days). Wound healing and blastema formation occur in day 1 and 2 post appendotomy (dpa), but by day 7 the leg bud is still very short, either as an elongated blastema with undifferentiated, tightly packed cells, or with a multilayered, incompletely differentiated epithelium (not shown). This stage resembles the very early stage of regeneration in specimens in late intermolt phases. In the latter case, when the specimens are closer to the next molt, usually the regeneration progresses much quicker and within a week it is close to completion (Figs. 1C, 3G and 5G). However, in other specimens at 32 dpm and 7 dpa, although the leg was elongated and coiled inside the coxa, it still had a multilayered epidermis, with muscle bundles that were incompletely developed, and showed no signs of cuticular formation yet (like in Fig. 5E). This suggests that the specimens were not as close to the next molt as predicted, thus highlighting the variance in molting cycle length among individuals.

### Anamorphic leg development

**Fig. 7:**
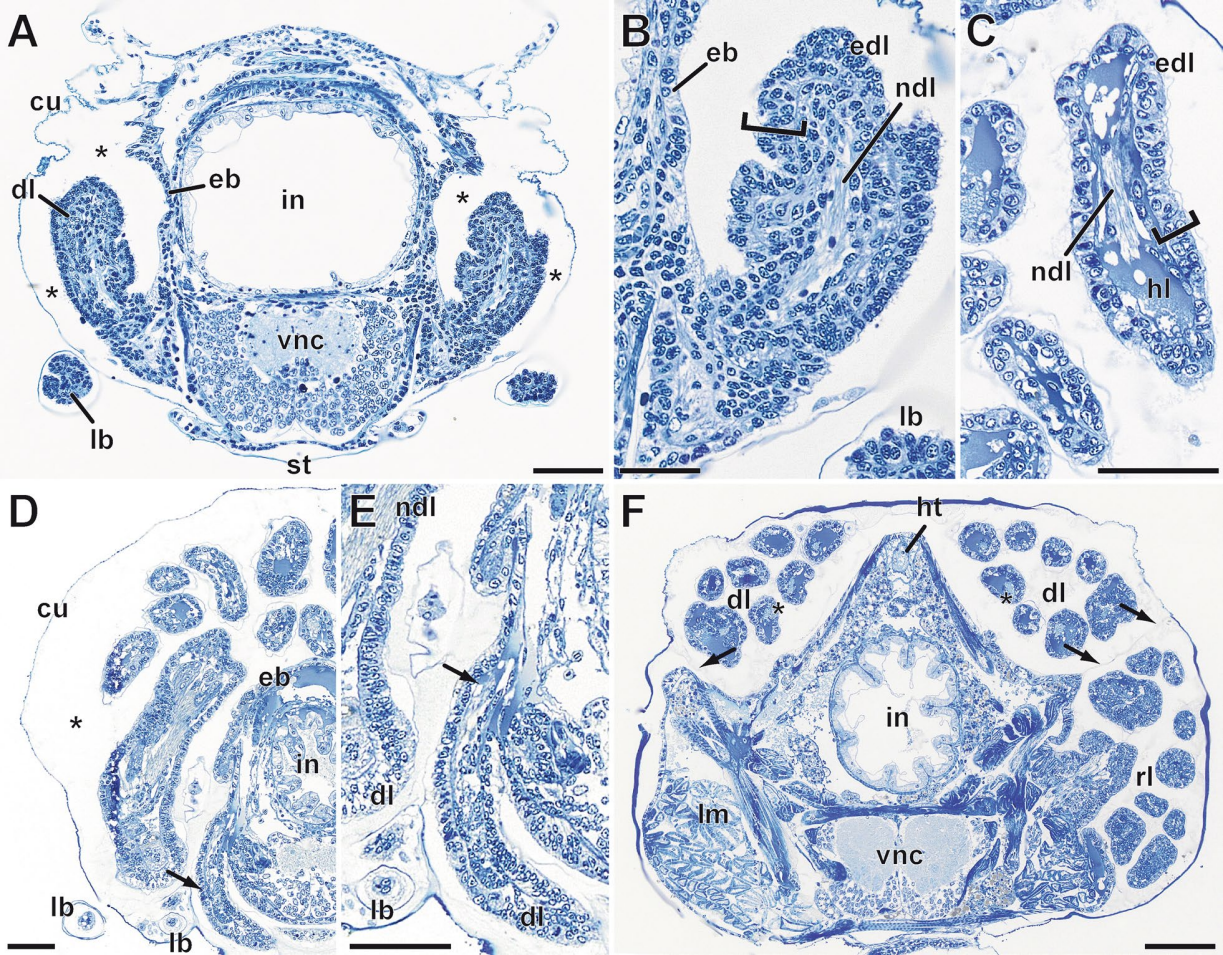
Histological aspects of developing legs in *Scutigera coleoptrata* instars. **A** Cross section of an instar IV in early interphase. Developing legs (leg 10 in this section) are present in the space between cuticle and the epidermis of the body wall (asterisks) and characteristically bend dorsad. Externally, developing legs possess small limb buds that will develop into tarsal elements. Scale bar = 50 µm. **B** Higher magnification of A. The epithelium of the developing leg is multilayered (bracket) and centrally innervated by a nerve. Scale bar = 25 µm. **C** Later stage of developing legs in an instar V in mid interphase. The epithelium of the developing leg is unilayered (bracket). The leg is centrally innervated by a nerve and filled with hemolymph. Scale bar = 50 µm. **D** Posterior cross section of an instar VI. Developing legs (legs 12 and 13) are coiled inside the lumen between cuticle and body wall (asterisk). Limb buds of developing legs are present ventrally. Medioventrally, the anlagen of the developing legs that will grow in the next intermolt are present (arrow), and characteristically bend ventrad. Scale bar = 50 µm. **E** Higher magnification of D. The anlage still has a multilayered epithelium, but is already innervated by a small nerve (arrow). Scale bar = 50 µm. **F** More anterior cross section of an instar V with developing legs (asterisks), and a regenerating leg in the right coxa. A thin membrane (arrows) separates the developing and regenerating legs. Compare also the coxa of the regenerating leg with the intact coxa. Scale bar = 100 µm. Abbreviations: cu cuticle, dl developing leg, eb epidermnis of the body wall, edl epidermis of developing leg, hl hemolymph, ht heart, in intestine, lb limb bud, lm locomotory leg musculature, ndl nerve of developing leg, rl regenerating leg, st sternite, vnc ventral nerve cord

*Scutigera coleoptrata* instars hatch with four leg bearing segments and grow by adding one leg-bearing segment after the first instar stage and two leg-bearing segments after the next 6 intermolt phases [31, 48]. The legs develop laterally on the posterior segments in a coiled manner under the cuticle and gradually extend dorsally and anteriorly as they grow (Figs. 6A–C and 7A, D). The legs progressively develop throughout the entire intermolt phase. Initially, they have a multilayered epidermis, and the lumen contains hemolymph and frequently myocytes. A nerve projecting from the respective segmental ganglion of the ventral nerve cord innervates each leg (Fig. 7A, B). In late stages of the intermolt phase, the epidermis is unilayered, and muscle bundles are present (Fig. 7F). Ventrolaterally, the distal portions of the developing legs form external buds (Figs. 6A–C and 7A, D). From the fourth instar stage onward, the last pair of legs resembles the ultimate legs of adult individuals. These legs are oriented posteriad, and the instars even exhibit the characteristic tapping behavior associated with the ultimate legs in adults. Histological analyses reveal that also anlagen of developing legs are present one molting cycle before they are observed coiled under the cuticle. Thus, posterior to the developing leg pairs that will emerge after the next molt, the anlagen of the legs of the next segments are already present (Fig. 7D, E arrow). The incompletely differentiated epidermis of these segments folds out laterally and forms the anlagen of the legs in a gap between the cuticle and the body wall (Fig. 7D, E). At this stage, the anlagen are already innervated by the nervous system, but only slightly increase in size during the intermolt phase. Only during the next intermolt phase, these leg anlagen start to grow and elongate, as well as coil and extend dorsally and anteriorly between the cuticle and the body wall (compare Fig. 7A–D).

As instars of *S. coleoptrata* can also lose and regenerate legs, we were able to observe the two leg formation processes in parallel in the same animal (Figs. 6A, B and 7F). Although the two processes are initiated by different factors (injury, respectively developmental program), the morphogenesis of the legs is very similar (compare Figs. 5, 6 and 7). Both regenerating and developing legs follow the same steps described above and grow in a spiral manner in a space between cuticle and body wall epidermis. However, the regenerating legs are limited to the space of the coxa of the injured leg (Figs. 6A, B and 7F), while the developing legs extend dorsally and anteriorly over multiple segments (Fig. 6A–C). Thus, if posterior legs are lost, the developing and regenerating legs grow next to each other (Figs. 6A, B and 7F) in the space between the body wall and the cuticle, separated only by a thin membrane (Fig. 7F).

## Discussion

### Losing legs: preferred breakage point and appendotomy

A preferred breakage point, which often facilitates appendotomy, is present in most hexapods and decapod crustaceans, as well as in several chelicerate and centipede species [1, 54]. However, the presence of a PBP does not imply that an arthropod species can effortlessly and without further injury lose a leg, as seen in the house centipede *Scutigera coleoptrata.* For example, in locomotory legs of *Lithobius* spp. (Chilopoda, Lithobiomorpha) a PBP is present between coxa and trochanter [55], however, their legs are more robust and much shorter than those of *S. coleoptrata*, and the PBP is passed through by multiple muscles, making appendotomy a much more strenuous task, which thus occurs very seldom [51, 55, 56]. Accordingly, it is intriguing that in different centipede taxa the position of a PBP is variable.

In most arthropods that can readily appendotomize their appendages, the PBP is accompanied by additional structures, which aid wound closure and prevent fluid loss after injury. In some crustaceans, hemolymph vessels are equipped with valves that close immediately after leg loss. Usually no muscles cross the PBP, however, some taxa possess an autotomizer muscle, which aids the mechanical detachment of the leg [54, 57, 58]. Moreover, a diaphragm which aids the wound closure has been documented in combination with a PBP in Decapoda, Isopoda, Odonata, Orthoptera and Phasmida, as well as in *S. coleoptrata* [1, 52]. In crayfish, the diaphragm is a single layer (“a connective-tissue membrane”) with small apertures for the blood vessels and the leg nerve [54]. Herbst [52] erroneously described the diaphragm to be located in the coxa of *S. coleoptrata.* This led to the assumption that the breakage plane is located between the coxa and the trochanter, an idea which was later taken on by Verhoeff [40]. However, Manton [51] correctly stated, as also proven in our analysis (Fig. 2B), that the breakage plane is located between the trochanter and the prefemur. Thus, the diaphragm in the trochanter in combination with the PBP in the legs of *S. coleoptrata* allows these animals to easily lose legs in order to escape predators.

Arthropod wound healing has been investigated in only a few species of decapod crustaceans and hexapods [17, 59–61]. Initially, hemocytes (granulocytes and plasmatocytes) gather at the wound site and release clotting enzymes, which coagulate the hemolymph and form a scab [62]. In *S. coleoptrata*, leg appendotomy is aided by hemolymph that quickly seals the wound. This species’ hemolymph has a remarkably strong coagulation property [63]. Hilken et al. [64] showed that the plasmatocytes of *S. coleoptrata* produce a fibrous material, which is transported out of the cells and most likely contributes to the particularly fast coagulation. The authors also proposed that this special coagulation property functionally correlates with this species' ability to appendotomize. However, in order to reveal the potential role of hemocytes in wound healing, a detailed TEM investigation is required.

A few hours after the scab is formed, it gets a dark pigmentation (Fig. 1C). This wound melanization process is common throughout arthropods [22, 65], and is associated with enzymatic cascades in the hemolymph as part of the immune response [62, 66, 67]. Although studies on myriapod wound healing and their innate immune system are very limited [64, 68], we assume that the process herein described in *S. coleoptrata* is similar to that in Pancrustacea.

### Replacing the loss: timing and limitations of regeneration

After appendotomy and scab formation, the epidermis underneath starts to undergo changes. As described in some Pancrustacea [22, 54, 58, 59, 69], the epidermis under the scab stretches and closes the wound internally, as also seen in *S. coleoptrata*. The next steps of regeneration follow the same sequence, but the time intervals differ. Such temporal variations and separation of the regeneration phases are common in arthropods [22, 58, 60, 70, 71]. Adiyodi [58] divided regeneration in *Paratelphusa hydrodromous* (Decapoda) in three main phases: basal limb growth, growth plateau, and premolt growth. The duration of those steps depends on the time in the molting cycle when the leg was appendotomized. In some decapod species, regeneration can even shorten the molting cycle considerably [16, 70, 72], whereas in cockroaches, for example, it can prolong it [73]. In *S. coleoptrata*, multiple leg loss only affects the length of the molting cycle if over 26 legs (out of 30) have been removed. These specimens shorten their molting cycle by half, while those with more than 4 legs left take just as many days as unharmed specimens [39].

As shown by Adiyodi and Adiyodi [74], timing and degree of tissue growth and differentiation are regulated by ecdysis hormones. Exogenous decrease in molt inhibiting hormones accelerate regeneration, but result in underdeveloped legs, whereas high levels of molting hormones inhibit regeneration either in very early post-molt stages [54] or after the critical point, in very late pre-molt stages [14]. Similar to regeneration in decapods, in *S. coleoptrata* the first growth phases are slowed down in post molt and early intermolt stages and are accelerated in the late intermolt and premolt stages. Furthermore, the different stages of regeneration observed in individuals of *S. coleoptrata* at identical timepoints post molt and post appendotomy highlight how intricate these regulatory networks are, and how variable the length of the molting cycle can be between individuals.

### Fantastic legs and where to find them: anamorphic development versus regeneration

Development in centipedes is categorized as either epimorphic or anamorphic. Epimorphs (Scolopendromorpha and Geophilomorpha) hatch with a complete number of leg bearing segments (21–191 segments) while anamorphs (Scutigeromorpha, Lithobiomorpha, and Craterostigmomorpha) have a variable number of instar stages through which segments are added after hatching. Scutigeromorpha have six instar stages, Lithobiomorpha five, while Craterostigmomorpha only have one anamorphic instar stage [31, 75–77]. While there is no evidence of regeneration in Craterostigmomorpha, stone centipedes (Lithobiomorpha) are capable of regenerating their antennae, forcipules, and locomotory legs [36–38]. Moreover, there is indirect evidence that giant centipedes (Scolopendromorpha) can regenerate their ultimate legs [35]. Species of Geophilomorpha, on the other hand, seem to only regenerate their forcipules [36, 78], while there is limited information available regarding leg and antennal regeneration in this taxon.

In *S. coleoptrata*, leg regeneration can occur simultaneously with anamorphic leg development. Nevertheless, the location of the two processes is different. The developing legs extend laterally and dorsally under the cuticle over multiple segments, some of which are not yet fully developed. The regenerating legs grow in the coxa of the affected leg. Moreover, the timing of the two processes differs. The developing legs always follow the same temporal pace of growth and differentiation throughout the molting cycle. On the other hand, the growth and tissue differentiation of regenerating legs throughout the molting cycle varies depending on the time of appendotomy.

Regeneration is always initiated by injury or tissue loss and is almost exclusively dependent on the presence of nervous innervation. Due to these distinct factors, there are regeneration-specific genetic pathways and specific enhancers [79–81]. Nonetheless, distinct growth factors and developmental patterning genes are present during regeneration as well [9, 82–84]. In crickets, for example, the molecular mechanisms underlying regeneration seem to at least partially recapitulate development [85]. However, like in many other arthropods, no matter where the leg was amputated, or how many molts have passed, the regenerated leg of juvenile crickets is still either smaller than the original one, or lacks sensory or locomotory structures [86].

In an attempt to understand the evolution of regeneration, this event is often associated with certain developmental types (such as asexual reproduction, metamorphosis, anamorphosis). However, the highly irregular occurrence of regeneration throughout the animal kingdom shows that this is not always the case. Although tremendous progress is made in understanding the complex pathways of this phenomenon, the evolutionary origins of regeneration are still not fully understood.

### Long legs and explosive regeneration

Scutigeromorpha are famously some of the fastest and most agile land arthropods. They are feisty predators highly dependent on their speed and feed on smaller arthropods like flies, crickets and spiders [87]. Multiple adaptations play key roles in *S. coleoptrata*'s speed. There is a considerable extension of the coxa into a functional unit together with the pleurocoxa, katopleura and anopleura (Fig. 2C). This gives stability to the coxa and thus provides leverage for its rocking motion, and is a prerequisite for fast running with long legs [51]. This functional adaptation of the coxa serves a secondary purpose as well and aids the explosive regeneration: once the muscles inside compact after appendotomy, the coxa provides plenty of space for the very long legs to regenerate within one molting cycle. Although arthropod appendages often regenerate within the coxopodite, respectively telopodite, in a coiled manner [41, 54], the coxal space provided in *S. coleoptrata* is exceptional.

Progressive regeneration, seen in scolopendromorph or lithobiomorph centipedes, seems to have little impact on them, since their locomotion is hardly affected by an incompletely regenerated leg [30]. Moreover, their legs are much shorter and more robust, and their PBP does not allow for such an effortless breakage of the telopodite. Considering the ease with which *S. coleoptrata* loses its legs, as well as its long lifespan (over 7 years in captivity), a progressive and incomplete regeneration, or the lack of, would very quickly render the animal “unfunctional”. It is conceivable that smaller leg stumps, as seen in progressive regeneration, would disrupt the metachronic wave pattern that the legs achieve during fast running [30] and significantly imbalance the animal during locomotion, while also hindering prey capture.

If explosive regeneration in land arthropods were to be associated with long legs, harvestmen should also feature it. However, although they have similarly long and slender legs and can appendotomize, they do not regenerate them at all [1, 19, 88]. The purpose of their long appendages is rather for slower locomotion through loose litter than for speed and prey capture. Moreover, harvestmen can rapidly compensate negative locomotory effects caused by leg loss through adjustments in stride and postural kinematics [19, 89]. Finally, their lifespan is considerably shorter than that of house centipedes, most species closing their life cycles after one year [88]. Considering the different life history and ecological niche of harvestmen, as well as their alternative strategy to deal with the appendotomy of their long legs, it is conceivable that the costs of regeneration overweigh its benefits, and escape from predation is more important than leg replacement. Moreover, evolution does not always follow the same path. The harvestmen evolved an adaptive locomotory mechanism to cope with the loss of their long legs in order to retain functionality, house centipedes on the other hand, seem to fit the adaptive regeneration hypothesis [2, 90]: firstly, the frequency of appendage loss in the field, followed by the impediment of locomotion and prey capture and thus fitness-related costs leading to death. Last but not least, house centipedes have the benefit of safe appendotomy, a high regeneration fidelity, and most importantly, the benefit of structure replacement by their explosive regeneration.

## Conclusion

Clearly, great legs come with great responsibility, and there is a crucial condition for maintaining a complex locomotory system operational: housekeeping. Long and flexible legs are needed for high-speed locomotion, however, they are fragile and can easily be damaged, which in consequence would result in an impairment of locomotion. Thus, long legs need to be easily detached in case of injury. But if they can easily be detached, they can easily “run out”. In the case of harvestmen, functionality of locomotion is still given and the biological value of predator escape might have a higher value as the replacement of lost legs (as discussed by [54]). Scutigeromorph centipedes, however, highly benefit from explosive regeneration, as this mode of leg replacement is ideal for an arthropod taxon, which, through its ecological niche, depends so greatly on all its legs, speed and agility. It is thus very advantageous to be able to permanently restore its locomotory system throughout its lifespan, and to do so as quickly as possible. Even more intriguing is the ability to lose and regenerate legs while simultaneously developing further leg bearing segments in anamorphic instars. The parallel observation of the two processes points out significant differences between them with respect to their timing and progression, but also a major overlap in the overall morphogenesis of the appendages. This, together with the points discussed above suggests that regeneration is likely a co-option in the developmental repertoire, and the differences are just a requirement for the novel context in which re-development occurs. However, its mode and “potency” are shaped by many more factors than we can understand today. *Scutigera coleoptrata*, with its anamorphic development, its appendotomy abilities and its explosive regeneration is thus a dazzling example of how fascinating, complex and multilayered adaptive evolution can be.

## Material and methods

### Animals and experiments

Adult individuals and instar stages of *Scutigera coleoptrata* (Linnaeus, 1758) were collected near Korneuburg (Austria) on Bisamberg. They were placed in separate boxes containing substrate collected from the same site and kept at ambient conditions throughout the year. The enclosures were sprayed with water on a weekly basis, and the animals were fed every two weeks with small crickets or *Drosophila* sp. flies. For a period of twelve months, the enclosures were regularly inspected to identify exuviae, record the timing of molting and determine the critical point in various stages. The regeneration experiments were performed in summer.

For all experiments, both instar stages and adult specimens were utilized (for individual experiments see below). Appendotomy was performed by gently holding the target leg with tweezers without exerting force. The immobilized legs were immediately left behind as the specimens continued to move. Legs were consecutively removed from the same specimens on different days to study the stages of regeneration at various time intervals (12 h, 1–10 days post-appendotomy—dpa) under identical conditions (for histological and immunhistochemical experiments). In most experiments, the legs of consecutive segments were removed from the same side (left or right). Neither the identity of the leg nor the regeneration of an abutting leg (same or opposite side) affected the progression of regeneration. The legs were removed from animals that were subsequently preserved while still in early stages of the intermolt period (10 days post-molt) or in late intermolt stages (18–22 days post-molt—dpm). The determination of early and late stages was based on the average duration of a molting cycle in late instar stages (stage VI) and pre-mature adults (which can still vary significantly) and by observations of the animals' appearance and behavior, which are typically associated with the approaching molt in terrestrial arthropods (e.g., changes in cuticle coloration, lethargy, and/or reduced appetite). Unfortunately, relying on external signs of premolt, particularly changes in behavior, proved to be ineffective in *S. coleoptrata*. The species did not exhibit consistent or traceable patterns of these typical changes. Often, specimens would molt overnight without exhibiting any external signs of premolt the day before, contradicting expectations based on the average duration of the molting cycle.

### Histology and light microscopy

For tissue fixation, four adult specimens and seven instars were immersed in FAE (a solution consisting of 37% formaldehyde, 80% ethanol, and glacial acetic acid in a ratio of 10:4:1) for 12–20 h (see experimental setup above) [91]. Subsequently, they were washed three times in phosphate-buffered saline (PBS; 0.1 M, pH 7.4) for 20 min. To achieve secondary tissue fixation, the samples were treated with 2% osmium tetroxide for 1 h and then washed three times in PBS for 20 min each. Following this, the samples underwent dehydration using an ascending series of acetone (30–90%, 3 × 100%) with each step lasting 20 min. Subsequently, the samples were placed in a 1:1 mixture of 100% acetone and low viscosity resin (Agar Scientific), covered with a lid, and left for 3 h. After removing the lid, the acetone was allowed to evaporate overnight. The following day, the samples were immersed in pure resin for 1 h, transferred to resin-filled embedding forms, and placed in a vacuum oven at 40 °C and 200 mbar for 20–60 min to eliminate any air bubbles. Subsequently, the samples were left in the oven at 60 °C overnight to allow the resin to polymerize. Alternatively, four specimens were fixed in fresh Karnovsky fixative (2.5% glutaraldehyde, 2% paraformaldehyde, 1.5% NaOH, and 1.5% D-glucose, buffered in 0.1 M PBS). Post-fixation in 2% OsO4 solution was conducted at room temperature for 1 h, followed by dehydration in a graded series of ethanol and embedding in Epon 812 resin (Serva). The resin blocks were manually trimmed and then serially sectioned using a Leica EM UC7 microtome (Leica Microsystems) with a DiATOME histo Jumbo knife at a thickness of 1 μm. The sections were transferred onto glass slides, stained with a solution of 1% toluidine blue in 1% borax at 60 °C, mounted in resin, and polymerized overnight at 60 °C. The semithin sections were examined and documented using a Nikon NiU compound microscope equipped with a Nikon DsRi2 camera, or a Olympus VS120 slide scanner. Images of whole mount samples were captured using a Nikon SMZ25 stereo microscope equipped with a Nikon Ds-Ri2 camera.

### Immunohistochemistry

For immunohistochemical experiments, three specimens (instars IV, V and VI) were examined (see experimental setup above). After anaesthetization by cooling down, the specimens were decapitated and fixed overnight in 4% paraformaldehyde in PBS. Specimens were washed in several changes of PBS, embedded in 4% agarose (Biozym LA Agarose, #840,004) in water, and horizontally sectioned (100 µm) using a Leica VT1000 S vibratome. Sections were preincubated in PBS-TX (PBS, 1% bovine serum albumin, 0.3% Triton X) for one hour at room temperature, and incubated in mouse anti-tyrosinated tubulin (1:1000, SigmaAldrich T9028) in PBS-TX overnight at room temperature. After several washing changes in PBS, they were then incubated in AlexaFluor 488 phalloidin (1:40, ThermoFisher A12379), anti-mouse AlexaFluor568 (1:1000, Invitrogen 11004) and bisbenzimide HOECHST 33342 (0.5 µg/ml, Invitrogen) in PBS for 4 h at room temperature. Finally, sections were washed for 2 h in several changes of PBS and mounted in Mowiol (Calbiochem). Analysis of the specimens was conducted using a Leica TCS SP5 II confocal laser scanning microscope as well as an Olympus Spinning Disc confocal microscope equipped with a Yokogawa unit (Microscopy facility at the Center for Cancer Research, Medical University of Vienna).

The monoclonal anti-tyrosine tubulin (mouse IgG3; Sigma Aldrich T9028, Clone TUB-1A2) was raised against a peptide containing the carboxy-terminal amino acids of α-tubulin. According to the manufacturer, this antibody reacts with tyrosine tubulin e.g. from bovine brain, kidney cells, yeast, and *Xenopus*, indicating that the antigen that this antibody recognizes is evolutionarily conserved across a broad range of species. In prior studies with *S. coleoptrata* this antibody was used for analyzing the nervous system [91]. Tubulin is the major building block of microtubules and represents a heterodimer of α- and β-tubulin. Tyrosinated tubulin represents a relatively dynamic subclass of interphase microtubules as tubulin tyrosinylation is involved in the assembly status of tubulin [92].

### Scanning electron microscopy

After anesthetization by cooling, several specimens were fixed in FAE (see above). After dissection and dehydration in a graded series of ethanol, preparations were transferred to glass vials and cleaned in an ultrasonic bath. Samples were critical-point-dried using the automated dryer Leica EM CPD300 and mounted on copper wire (Plano #16067) or carbon-conducted tabs (Plano #G3347) and finally sputter-coated with gold and examined with a Zeiss EVO LS10 (Imaging Center of the Department of Biology, University of Greifswald).

### MicroCT analysis

Two instar specimens (instar III, intact; and instar V, left leg 11 detached, fixed at 7 days post appendotomy) were fixed overnight using Bouin's fixative [93]. After fixation, the samples were washed three times in PBS for 20 min each. To dehydrate the samples, a graded series of ethanol solutions (50–90%, 3 × 100%) was used. Subsequently, the samples were incubated in 1% iodine in pure ethanol for 8 h and then washed several times in pure ethanol. After dehydration, the samples were mounted in plastic pipette tips using pure ethanol and sealed with hot glue. MicroCT scans were performed using a Zeiss XRadia XCT-200 (Department of Evolutionary Biology, University of Vienna). TIFF image stacks were obtained by reconstructing the tomographies in the XMReconstructor software (Zeiss Microscopy). The microCT data was then analyzed and reconstructed using Amira software (ThermoFisher).

## Data Availability

Data generated and/or analyzed during the current study are available from the corresponding authors on reasonable request.
